# A Salt-Signaling Network Involving Ethylene, Extracellular ATP, Hydrogen Peroxide, and Calcium Mediates K^+^/Na^+^ Homeostasis in *Arabidopsis*

**DOI:** 10.3390/ijms21228683

**Published:** 2020-11-17

**Authors:** Tao Lang, Chen Deng, Jun Yao, Huilong Zhang, Yin Wang, Shurong Deng

**Affiliations:** 1 Institute of Ecology, College of Urban and Environmental Sciences and Key Laboratory for Earth Surface Processes of Ministry of Education, Peking University, Beijing 100871, China; langt@pku.edu.cn; 2 State Environmental Protection Key Laboratory of Regional Eco-process and Function Assessment, Chinese Research Academy of Environmental Sciences, Beijing 100012, China; 3 Beijing Advanced Innovation Center for Tree Breeding by Molecular Design, College of Biological Sciences and Technology, Beijing Forestry University, Beijing 100083; China; deng51@bjfu.edu.cn (C.D.); yaojun@bjfu.edu.cn (J.Y.); hlzhang@caf.ac.cn (H.Z.); 4 State Key Laboratory of Tree Genetics and Breeding, The Research Institute of Forestry, Chinese Academy of Forestry, Beijing 100091, China

**Keywords:** ethylene, eATP, H_2_O_2_, [Ca^2+^]_cyt_, NaCl, K^+^/Na^+^ homeostasis, *Arabidopsis*

## Abstract

This work aimed at investigating the interactive effects of salt-signaling molecules, i.e., ethylene, extracellular ATP (eATP), H_2_O_2_, and cytosolic Ca^2+^ ([Ca^2+^]_cyt_), on the regulation of K^+^/Na^+^ homeostasis in *Arabidopsis* *thaliana*. The presence of eATP shortened Col-0 hypocotyl length under no-salt conditions. Moreover, eATP decreased relative electrolyte leakage and lengthened root length significantly in salt-treated Col-0 plants but had no obvious effects on the ethylene-insensitive mutants *etr1-1* and *ein3-1eil1-1*. Steady-state ionic flux kinetics showed that exogenous 1-aminocyclopropane-1-carboxylic acid (ACC, an ethylene precursor) and eATP-Na_2_ (an eATP donor) significantly increased Na^+^ extrusion and suppressed K^+^ loss during short-term NaCl treatment. Moreover, ACC remarkably raised the fluorescence intensity of salt-elicited H_2_O_2_ and cytosolic Ca^2+^. Our qPCR data revealed that during 12 h of NaCl stress, application of ACC increased the expression of *AtSOS1* and *AtAHA1*, which encode the plasma membrane (PM) Na^+^/H^+^ antiporters (SOS1) and H^+^-ATPase (H^+^ pumps), respectively. In addition, eATP markedly increased the transcription of *AtEIN3*, *AtEIL1*, and *AtETR1*, and ACC treatment of Col-0 roots under NaCl stress conditions caused upregulation of *AtRbohF* and *AtSOS2*/3, which directly contribute to the H_2_O_2_ and Ca^2+^ signaling pathways, respectively. Briefly, ethylene was triggered by eATP, a novel upstream signaling component, which then activated and strengthened the H_2_O_2_ and Ca^2+^ signaling pathways to maintain K^+^/Na^+^ homeostasis under salinity.

## 1. Introduction

Salinity, a typical abiotic stress, poses a major threat to the growth and development of herbaceous and woody species worldwide [1,2]. Salt injury causes direct disturbances in plant ionic balance. Moreover, in NaCl-treated plants, excessive sodium and chloride ions easily cause secondary stresses, such as oxidative stress and cytomembrane system damage [3,4]. To counter the disruption in ionic homeostasis resulting from cytosolic Na^+^ accumulation, Na^+^ extrusion and K^+^ retention in the overall plant and at the intracellular level play a pivotal role in adapting to salt injury [4,5]. Na^+^/H^+^ antiporter and H^+^-ATPase, two functional proteins located in the plasma membrane (PM) of higher plants, are indispensable for maintaining K^+^/Na^+^ homeostasis [6,7,8,9,10]. Specifically, PM SOS1 helps to retain superfluous Na^+^ in cytoplasts [3,11]. The PM H^+^ pump drives Na^+^ and H^+^ antiport via maintenance of an H^+^ electrochemical gradient under salt conditions [12]. Besides controlling Na^+^ homeostasis, the upregulation of PM H^+^ pumps effectively protects the less-depolarized membrane potential and, thus, restricts salt-elicited K^+^ loss (efflux) through outward rectifying depolarization-activated non-selective cation channels (DA-NSCCs) and K^+^-permeable channels (DA-KORCs) [9,13].

Industrial production of ethylene (ET) is said to be one of the highest of all chemical products produced worldwide [14]. Naturally produced ethylene also serves as a diffusible phytohormone that regulates various aspects of plant development and stress responses [15]. In *Arabidopsis*, ethylene-related signaling transduction is a complicated process that regulates downstream physiological, biochemical, and even molecular responses [16,17,18]. In concrete terms, ethylene binding appears to inactivate the constitutive triple response 1 (CTR1, a negative regulator) kinase; however, CTR1 can be activated by an unoccupied ethylene receptor, such as ethylene response 1 (ETR1, an ethylene receptor), leading to a physiological interaction. Subsequently, CTR1 activates transcriptional factors ethylene insensitive 2/3 (EIN2/3) and ethylene insensitive-like 1 (EIL1) to mediate downstream signaling pathways [19,20]. EIN3 and EIL1 play important roles in regulating the ethylene signaling pathways that are activated via EIN2, and are necessary for the transcriptional induction of target ethylene response genes, such as ethylene response factor 1 (ERF1, also called ESE1), thereby modulating multiple responses in plants [21,22,23]. The process of ethylene signaling transduction markedly affects plant salt tolerance [18]. Some evidence shows that salt first induces a quick marked rise in ethylene [24], and the subsequent application of exogenous 1-aminocyclopropane-1-carboxylic acid (ACC, an ethylene precursor) can dramatically improve salt tolerance in *Arabidopsis* [21,25]. The germination rate of ethylene-insensitive *etr1-1* seedlings is significantly lower than that of Col-0 under high salinity [26]. Excessive accumulation of EIN3 is induced via NaCl treatment, and the *ein3eil1* mutant is oversensitive to salt stress [25]. In summary, signal transduction is vital to the salt response in plants. However, the mechanism of how ethylene contributes to the regulation of K^+^/Na^+^ homeostasis under saline conditions has not been precisely elucidated in higher plants.

Besides ethylene, diverse past studies have demonstrated that other crucial salt-sensitive signaling molecules in plants, such as eATP, H_2_O_2_, and Ca^2+^, and their interactions, are involved in the regulation of PM SOS1 and H^+^ pumps for the maintenance of ionic homeostasis under NaCl stress [7,8,10,13,27,28,29,30]. Cytosolic Ca^2+^ ([Ca^2+^]_cyt_) alleviates Na^+^ toxicity by activating the PM Na^+^/H^+^ antiport system in various plant species [4,7,10,30,31]. H_2_O_2_, an important reactive oxygen species (ROS) in plants, acts as a critical signal to trigger the salt overly sensitive (SOS) signaling pathway [28,29]; the subsequently entry of Ca^2+^ through PM Ca^2+^-permeable channels prompts Na^+^ exclusion and limits K^+^ leakage under high-salinity conditions [32,33]. In addition, [Ca^2+^]_cyt_ maintains ionic homeostasis via increased H_2_O_2_ in both secretor and non-secretor mangroves under high-salt stress [8]. Recently, Lang et al. (2017) showed that H_2_O_2_ interacts with [Ca^2+^]_cyt_-induced salt-responsive genes, e.g., *GuSOS3*/*GuCIPK*, in the perennial herb *Glycyrrhiza uralensis* during NaCl stress, and these genes are involved in the Ca^2+^‒SOS signaling pathway [10]. eATP, which is sensed by a purinergic receptor named P2 in the PM (likely P2K1 in higher plants [34]), functions in resisting salt injury [2,8,10,30,35]. Indeed, it has been proposed that salt-inducible eATP signaling triggers downstream signaling components of the salt response, such as [Ca^2+^]_cyt_ and ROS [36,37]. In the eATP-induced mechanism of salt resistance, eATP positively regulates the transcription of salt-related genes, e.g., *SOS1/3*, *RbohD* (respiratory burst oxidase homolog protein D, one of the NADPH oxidases that catalyze production of ROS), and *MAPK3/6* (mitogen-activated protein kinases 3 and 6), which trigger the Ca^2+^‒SOS, H_2_O_2_‒Rboh, and ATP‒MAPK signaling pathways, respectively [10]. Interestingly, ethylene also interacts with [Ca^2+^]_cyt_ and H_2_O_2_ during NaCl exposure. For example, the expression of *ERF1*, an ethylene-responsive target gene, is activated by *SOS2*, and contributes to the Ca^2+^‒SOS signaling pathway in *Arabidopsis* [38]. Similarly, recent studies have found that ethylene is involved in Ca^2+^-regulated Na^+^ homeostasis in adventitious roots of cucumber subjected to salt stress [39,40]. Moreover, ethylene efficiently sustains K^+^ homeostasis under high salinity via assistance from another NADPH oxidase, RbohC [41]. However, there is no evidence clearly indicating whether ethylene interacts with eATP to sustain ionic homeostasis upon NaCl stress. Hence, in this study, we sought to address two questions that have yet to be answered in higher plants: (1) whether ethylene interacts with eATP in salt-treated plants; and (2) how a novel salt-resistant signaling pathway related to ethylene, eATP, Ca^2+^, and H_2_O_2_ positively sustains ionic homeostasis under saline conditions.

In the present study, to determine whether eATP interacts with ethylene to contribute to salt tolerance, we examined the phenotypes of eATP-treated *Arabidopsis* Columbia (Col-0) and the ethylene-sensitive mutants of *etr1-1* and *ein3-1eil1-1*, such as root length, hypocotyl length, and relative electrolytic leakage, under short-term salinity treatment. Using non-invasive micro-test technology (NMT), we investigated the changes in steady Na^+^, K^+^, H^+^, and Ca^2+^ fluxes in *Arabidopsis* roots in the presence or absence of certain factors relevant to this study and with NaCl treatment. Furthermore, we screened the crosstalk among the various salt-sensitive signaling pathways by laser scanning confocal microscopy (LSCM) and examined the expression of genes related to K^+^/Na^+^ homeostasis by real-time quantitative PCR (qPCR). Our aim was to establish a multiple signaling network of ethylene, eATP, Ca^2+^, and H_2_O_2_ in the maintenance of ionic homeostasis in higher plants under salt stress.

## 2. Results

### 2.1. Influences of eATP on Ethylene-Responsive Seedling Phenotype and Electrolyte Leakage with or without Salinity

**Figure 1:**
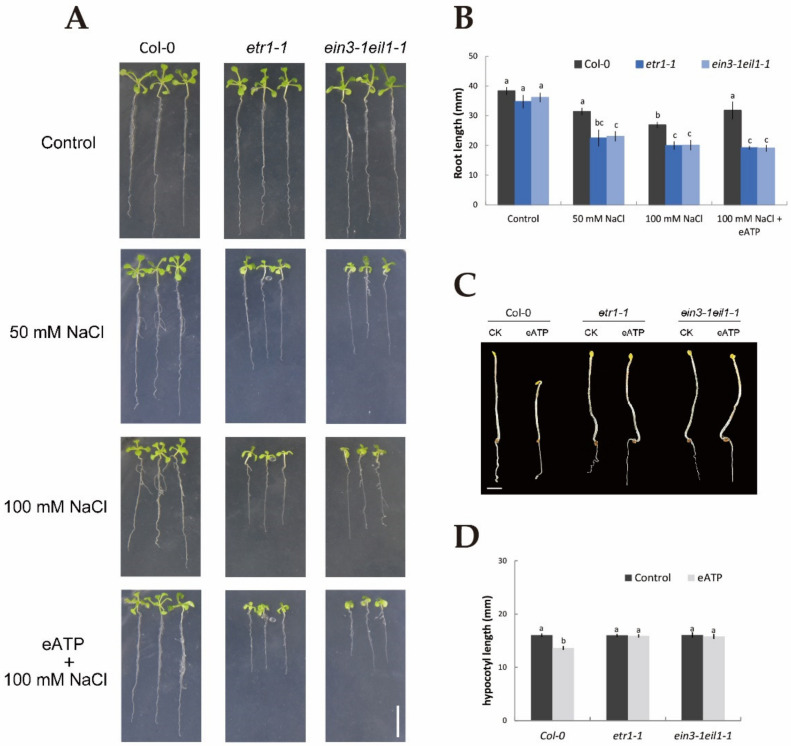
Application of extracellular ATP results in alterations of phenotypic identification, root length, and hypocotyl length in Col-0, *etr1-1*, and *ein3-1eil1-1* plants in the presence or absence of salinity. (**A**) Seeds were sown on 1/2 MS (Murashige and Skoog) medium; after germination, plants were transferred to media and subjected to 50 or 100 mM NaCl in the presence or absence of 300 μM ATP-Na_2_ (eATP), with control plants cultured in 1/2 MS NaCl-free (0 mM NaCl) medium. The root length was measured after 10 days. Scale bars, 10 mm. (**C**) Col-0, *etr1-1*, and *ein3-1eil1-1* seeds were sown on 1/2 MS medium with dark treatment in the presence or absence of 300 μM eATP. After germination, these seedlings were screened by a stereoscope and photographed. Scale bar, 2 mm. (**B**,**D**) quantification of the root and hypocotyl lengths, as depicted in (**A**,**C**). Each column (± SD) represents the mean of five to six individual plants, and the letters a, b, and c denote significant differences between different treatments (*p* < 0.05).

To determine whether ethylene and eATP could respond to NaCl and whether ethylene interacted with eATP in terms of the resulting phenotype and physiological changes, parameters, such as root length, relative electrolyte leakage, and hypocotyl length, were examined in Col-0, *etr1-1*, and *ein3-1eil1-1* with or without salt stress treatment. In the absence of NaCl stress, 10-day-old seedlings of Col-0, *etr1-1*, and *ein3-1eil1-1* showed no obvious difference in phenotype (Figure 1A). However, under 50 mM NaCl, the root length was visibly shortened by 22% in Col-0, 55% in *etr1-1*, and 57% in *ein3-1eil1-1*, and more dramatically by 43%, 74%, and 80% under 100 mM salt exposure (Figure 1A,B). Interestingly, under 100 mM salt treatment, the addition of 300 μM eATP resulted in an 18% increase in the root length of Col-0 (Figure 1A,B) but no marked changes in *etr1-1* and *ein3-1eil1-1*. We then compared the salt-induced relative electrical conductivity of wild-type and mutant seedlings with and without eATP. Salt stress induced a dramatic increase in relative electrolyte leakage in the ethylene-insensitive mutants compared with Col-0 plants; a similar result was found for eATP, which played a positive role in decreasing the relative electrolyte leakage elicited by 100 mM NaCl in Col-0 plants (down by around 25%, Figure S1) compared to no obvious change in the ethylene-insensitive mutants. In addition, eATP obviously shortened the hypocotyl length in the Col-0 background (by around 18%) but not ethylene-insensitive mutant seedlings (Figure 1C,D). Figure 1 and Figure S1 suggest that ethylene and eATP play a beneficial role in NaCl-treated plants, such as through promoting growth and blocking ion leakage. Furthermore, ethylene was involved in the eATP signaling pathway both in the presence or absence of salt conditions.

### 2.2. Salt-Elicited Alterations of Na^+^, K^+^, H^+^, and Ca^2+^ Fluxes in Roots

**Figure 2:**
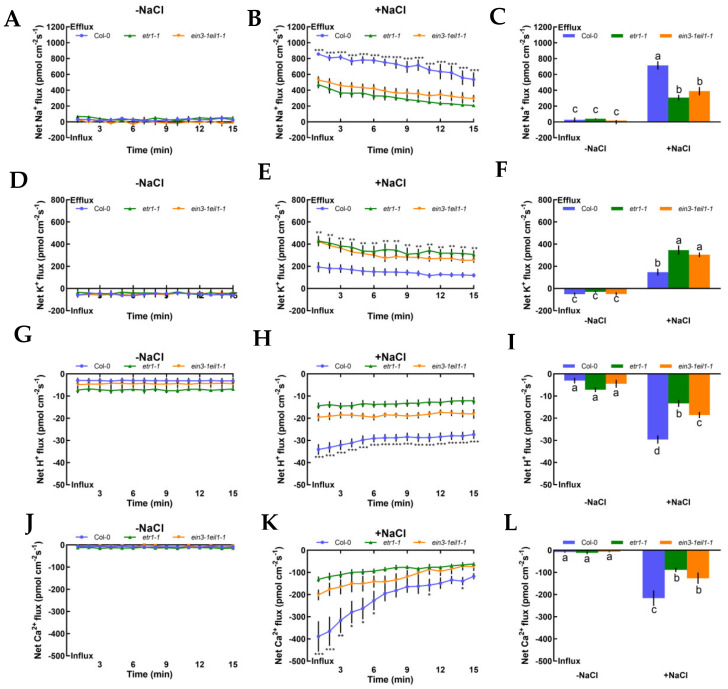
Effects of short-term NaCl stress (100 mM, 12 h) on the steady-state Na^+^, K^+^, H^+^, and Ca^2+^ flux kinetics in Col-0, *etr1-1*, and *ein3-1eil1-1* roots. Young roots were cultured in MS medium without (−NaCl) or with NaCl (100 mM) for 12 h. Steady-state Na^+^ (**A**‒**C**), K^+^ (**D**‒**F**), H^+^ (**G**‒**I**), and Ca^2+^ (**J**‒**L**) flux kinetics were recorded at the meristem root zones (500 μm from the root apex) in salt-untreated (left panels) or salt-treated roots (middle panels) for 15 min (compared to control groups, * *p* < 0.05, ** *p* < 0.01, and *** *p* < 0.001). Columns in the right panels represent the mean of each net ion flux at all salt-untreated (‒NaCl, without NaCl, the same below) and salt-treated (+NaCl, with NaCl, the same below) time points and the letters a, b, c, and d denote significant differences between strains (*p* < 0.05). Each point or column (± SD) represents the mean of five to six individual plants.

We also investigated steady-state Na^+^, K^+^, H^+^, and Ca^2+^ flux kinetics in the presence or absence of NaCl stress in Col-0, *etr1-1*, and *ein3-1eil1-1* roots to determine whether ethylene could produce responses to salt in terms of ionic alterations. Under no-salt treatment, Col-0, *etr1-1*, and *ein3-1eil1-1* exerted stable constant Na^+^ efflux in the root meristematic zones (500 μm from the root apex), with a mean rate of 28.14, 40.15, and 14.06 pmol cm^−2^ s^−1^, respectively (Figure 2A‒C; positive values denote ion efflux, while negative values denote influx, and the same applies below). After 12 h of 100 mM NaCl stress, the highest Na^+^ efflux was observed in Col-0 and the mean rate increased significantly to 713.75 pmol cm^−2^ s^−1^ (2.3- or 1.8-fold higher than that of salt-treated *etr1-1* or *ein3-1eil1-1*). The efflux rate gradually declined over time, with the exception of a few test points. However, the opposite results were found in salt-induced K^+^ leakage in meristematic zones of control and mutants in response to NaCl stress, wherein the salt-triggered K^+^ efflux in Col-0 decreased by 57.3% or 51.4% compared to that of *etr1-1* or *ein3-1eil1-1* (Figure 2D‒F). Salt-elicited H^+^ exhibited a remarkable shift in influx kinetics during 15 min of continuous testing, which indicates the activation of PM Na^+^/H^+^ antiport system activity (Figure 2G‒I). Compared with no-salt treatment, both Col-0 and mutant roots exerted vigorous Ca^2+^ influx kinetics with the highest rate of 215.89 pmol cm^−2^ s^−1^ (Figure 2J‒L). In Figure 2, salt caused cellular ionic disorders, such as Na^+^ efflux, K^+^ efflux, H^+^ influx, and Ca^2+^ influx; meanwhile, *etr1-1* and *ein3-1eil1-1* had a lower capacity to sustain ion homeostasis compared with Col-0 plants.

**Figure 3:**
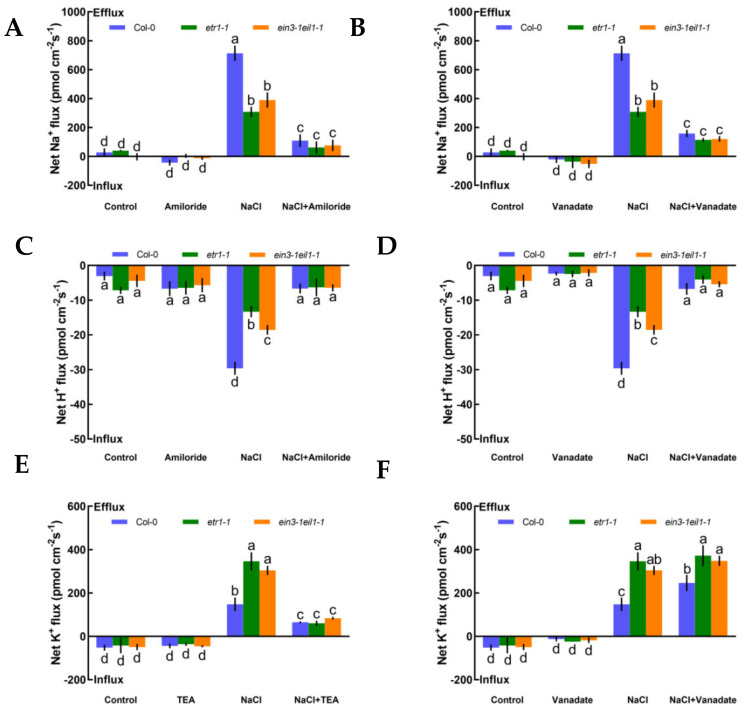
Pharmacological effects of amiloride (50 μM), sodium orthovanadate (500 μM), and tetraethylammonium (TEA, 50 μM) on the steady-state Na^+^, K^+^, and H^+^ flux kinetics in Col-0, *etr1-1*, and *ein3-1eil1-1* roots under short-term NaCl stress (100 mM, 12 h). Young roots were cultured in MS medium without (−NaCl) or with NaCl (100 mM) for 12 h, then subjected to treatment with ion channel-related inhibitors for 30 min prior to the determination of flux kinetics. Salt-elicited Na^+^ (**A**,**B**), H^+^ (**C**,**D**), and K^+^ (**E**,**F**) flux kinetics were recorded at the meristem root zones (500 μm from the root apex) in the absence (left) and presence (right) of ion channel-related inhibitors for 15 min. Each column (± SD) represents the mean of five to six individual plants and the letters a, b, c, and d denote significant differences between different treatments (*p* < 0.05).

To evaluate whether K^+^, Na^+^, and H^+^ fluxes were connected with H^+^-ATPase and specific ion channels, such as Na^+^/H^+^ antiporter and DA-KORCs, we measured the effects of specific ion transporter/channel inhibitors in the PM to measure the sensitivity of K^+^, Na^+^, and H^+^ channels to salt stress. In the absence of NaCl treatment, K^+^, Na^+^, and H^+^ fluxes showed no clear changes after the application of amiloride, sodium orthovanadate, and tetraethylammonium (TEA), specific blockers of the PM Na^+^/H^+^ antiporter, H^+^-ATPase, and the series of known K^+^ channels DA-KORCs, respectively (Figure 3). However, salt-triggered Na^+^ and H^+^ flux kinetics in roots were simultaneously suppressed by amiloride and sodium orthovanadate (Figure 3A‒D). Similarly, application of a known K^+^ channel blocker, TEA, resulted in dramatic reductions in NaCl-induced K^+^ efflux by 2.3-fold in Col-0, 5.7-fold in *etr1-1*, and 3.6-fold in *ein3-1eil1-1* (Figure 3E). In terms of the effect of the PM H^+^-ATPase inhibitor on the K^+^-permeable channel, the NaCl-induced K^+^ loss in Col-0 roots was significantly strengthened by sodium orthovanadate, but there were no obvious effects in *etr1-1* and *ein3-1eil1-1* (Figure 3F). As shown in Figure 3, these results indicate that salt-elicited Na^+^ efflux and H^+^ influx were mediated by the PM Na^+^/H^+^ antiporter, and K^+^ efflux was mediated by DA-KORCs; meanwhile, NaCl activated the activity of H^+^-ATPase, which offers energy for ion transportation.

**Figure 4:**
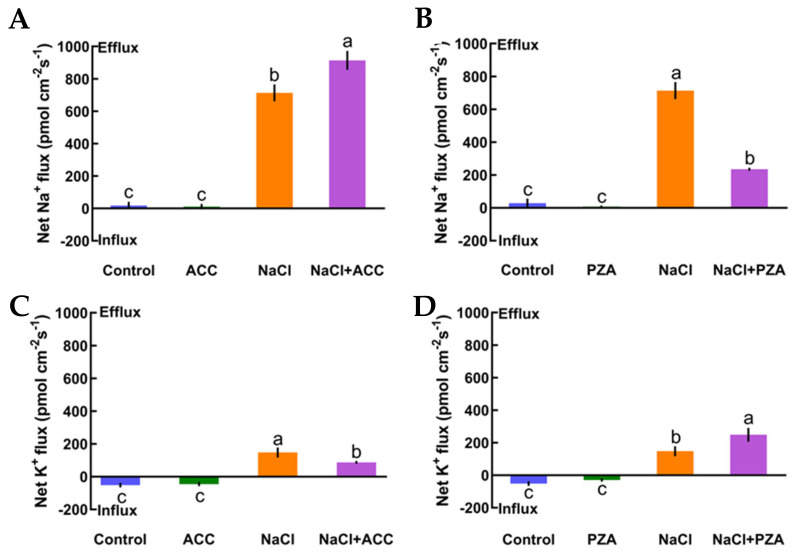
Effects of 1-aminocyclopropanecarboxylic acid (ACC, 10 μM) and pyrazinamide (PZA, 100 μM) on the steady-state Na^+^ and K^+^ flux kinetics of Col-0 roots under short-term NaCl stress (100 mM, 12 h). Roots cultured in MS medium were untreated or subjected to short-term NaCl stress (100 mM, 12 h) in the absence or presence of ACC (10 μM). For the pharmacological inhibitor experiments, MS-cultured roots were untreated or stressed by NaCl for 12 h, then subjected to PZA (100 μM) for 30 min prior to measurement of flux kinetics. Salt-elicited Na^+^ (**A**,**B**) and K^+^ (**C**,**D**) flux kinetics were recorded at the meristem root zones (500 μm from the root apex) in the absence and presence of ACC or PZA. Each column (± SD) represents the mean of five to six individual plants and the letters a, b, and c denote significant differences between different treatments (*p* < 0.05).

In this section, we offer direct evidence that ethylene maintains K^+^/Na^+^ homeostasis in high-salt conditions. First, under no-salt conditions, the application of exogenous ACC (an ethylene precursor, 10 μM) and pyrazinamide (PZA, a specific blocker of ethylene receptor, 100 μM) exhibited obvious effects on the steady state of K^+^ and Na^+^ fluxes in Col-0 plants (Figure 4A‒D). However, in the presence of short-term salt treatment, the application of exogenous ACC increased Na^+^ exclusion by 28% in the Col-0 root meristematic zones (Figure 4A). Conversely, the addition of exogenous PZA significantly decreased salt-elicited Na^+^ flux kinetics in Col-0 roots (Figure 4B). Moreover, the addition of ACC reduced K^+^ leakage by 69% in salinized Col-0 roots, whereas the opposite effect was seen with the application of a specific antagonist, PZA, in salt-treated Col-0 roots (Figure 4CD). The results in Figure 4 indicate that the ethylene functions in sustaining K^+^/Na^+^ homeostasis in Col-0 plants under saline stress.

**Figure 5:**
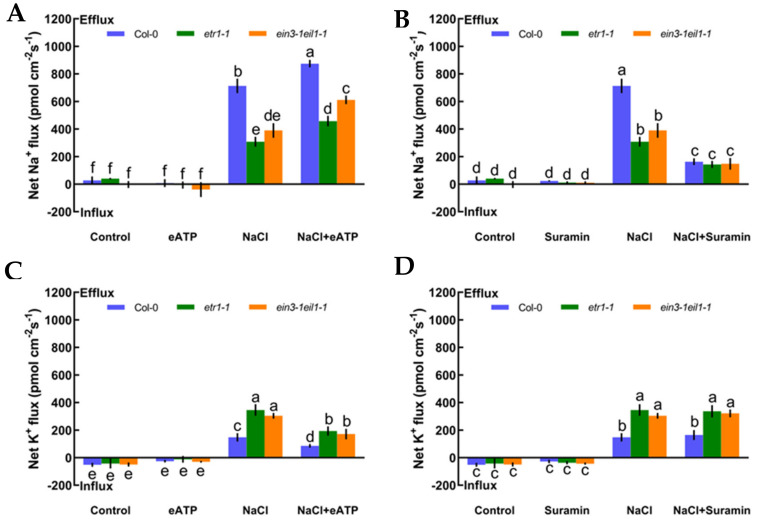
Effects of extracellular ATP (eATP, 300 μM) and suramin (300 μM) on the steady-state Na^+^ and K^+^ flux kinetics in Col-0, *etr1-1*, and *ein3-1eil1-1* roots under short-term NaCl stress (100 mM, 12 h). MS-cultured roots were untreated or subjected to short-term NaCl stress (100 mM, 12 h) in the absence or presence of eATP (300 μM). For pharmacological inhibitor experiments, MS-cultured roots were untreated or stressed by NaCl for 12 h, then subjected to suramin (300 μM) for 30 min prior to measurement of flux kinetics. Salt-elicited Na^+^ (**A**,**B**) and K^+^ (**C**,**D**) flux kinetics were recorded at the meristem root zones (500 μm from the root apex) in the absence and presence of eATP or suramin. Each column (± SD) represents the mean of five to six individual plants and the letters a, b, and c denote significant differences between different treatments (*p* < 0.05).

As described in Figure 4, we also measured whether eATP could maintain K^+^/Na^+^ homeostasis and interact with ethylene in salt-treated Col-0, *etr1-1*, and *ein3-1eil1-1*. First, we confirmed that eATP (300 μM) and suramin (an inhibitor of eATP, 300 μM) had no clear influences on ionic homeostasis in no-NaCl-treated *Arabidopsis* (Figure 5A–D). However, eATP enhanced the steady-state Na^+^ flux profiles in salinized Col-0, *etr1-1*, and *ein3-1eil1-1*. Furthermore, the enhancement of Na^+^ flux in Col-0 was 1.9-fold higher than that of *etr1-1* and 1.4-fold higher than that of *ein3-1eil1-1* under salt exposure (Figure 5A). However, with the addition of suramin, an ATP antagonist, the salt-triggered Na^+^ flux kinetics were dramatically reduced, with mean values of 163.37 pmol cm^−2^ s^−1^ in Col-0, 143.24 pmol cm^−2^ s^−1^ in *etr1-1*, and 147.86 pmol cm^−2^ s^−1^ in *ein3-1eil1-1*, respectively (Figure 5B). Under 100 mM salt treatment conditions, the capacity for restraining K^+^ leakage in eATP-treated Col-0 roots was much higher than that of the ethylene-insensitive mutants (Figure 5C). The addition of suramin slightly affected the salt-induced K^+^ flux kinetics under salt stress (Figure 5D). These results indicated that under salt stress, eATP played a vital role in terms of restricting K^+^ leakage and promoting Na^+^ exclusion in Col-0 and ethylene-insensitive mutants. However, the ability to maintain K^+^/Na^+^ homeostasis in ethylene-insensitive mutants was obviously lower than that of Col-0 both with and without salt, as there might be other signaling pathways independent of ethylene that can ease salt injury.

### 2.3. Ethylene-Induced Accumulation of ROS and [Ca^2+^]_cyt_ under NaCl Stress

**Figure 6:**
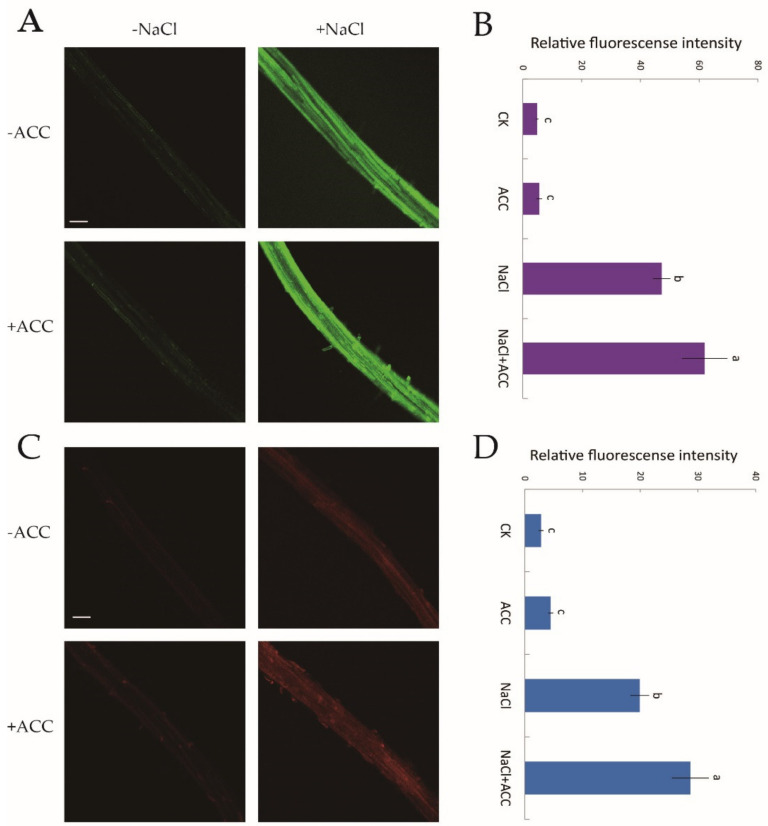
Effects of exogenous ACC on cytosolic H_2_O_2_ and Ca^2+^ accumulation in NaCl-exposed Col-0 root tips. Half of MS-cultured roots were untreated or subjected to a short-term NaCl stress (100 mM, 12 h) in the absence or presence of ACC (10 μM). Roots were then incubated with two specific fluorescent probes (H_2_DCF-DA, green and Rhod-2 AM, red) for H_2_O_2_ and Ca^2+^ detection, respectively, for 30 min. (**A**,**C**) Confocal images of H_2_O_2_ and Ca^2+^ fluorescence within root cells (scale bars, 250 µm); (**B**,**D**) the relative green and red fluorescence intensity at the maturation root zones (1000 μm from the root apex) with or without the application of ACC in salt-treated roots. Each column (± SD) represents the mean of five to six individual plants and the letters a, b, and c denote significant differences between different treatments (*p* < 0.05).

There is a cytosolic H_2_O_2_ and Ca^2+^ burst when plants are subjected to NaCl treatment [10]. However, whether ethylene upregulates H_2_O_2_ and [Ca^2+^]_cyt_ levels under salt stress requires confirmation. Hence, we used two specific fluorescent probes, H_2_DCF-DA and Rhod-2 AM, for the detection of cytosolic H_2_O_2_ and Ca^2+^, respectively. Confocal assays showed that under no-salt conditions, ethylene had little influence on H_2_O_2_ and [Ca^2+^]_cyt_ fluorescence (Figure 6A–D). However, the fluorescence intensity of NaCl-induced H_2_O_2_ and [Ca^2+^]_cyt_ was significantly boosted (by 31–44%) compared to control samples after exogenous ACC (10 μM, 30 min) shock (Figure 6B,D). The above results suggested that, under salinity stress, ethylene helped to mediate ROS and cytosolic Ca^2+^ signaling cascades.

### 2.4. Extracellular ATP- and Ethylene-Elicited Alterations in the Expression of Some Salt-Related Genes

**Figure 7:**
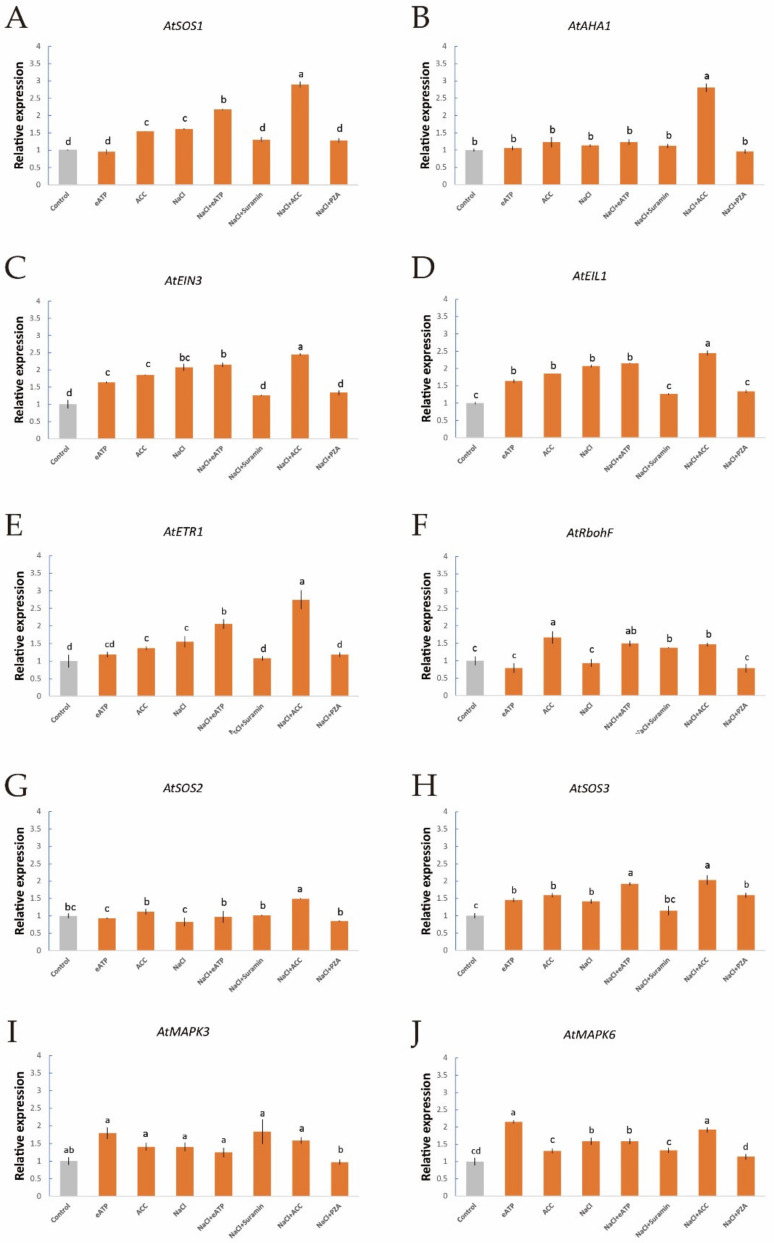
Effects of ACC and eATP on salt-responsive gene transcription of Col-0 roots under salt stress. Roots were exposed to 0 or 100 mM NaCl for 12 h, supplemented with or without ACC (10 µM) or ATP-Na_2_ (300 µM). Quantitative RT-PCR results show the relative transcript abundance of homolog genes in Col-0, such as (**A**) *AtSOS1* (the PM salt overly sensitive 1/(Na^+^/H^+^) antiporter), (**B**) *AtAHA1* (the PM H^+^-ATPase), (**C**) *AtEIN3* (the ethylene insensitive 3 gene), (**D**) *AtEIL1* (the EIN3-like 1 gene), (**E**) *AtETR1* (the ethylene response 1 gene), (**F**) *AtRbohF* (the respiratory burst oxidase homolog protein F gene), (**G**) *AtSOS2* (the salt overly sensitive 2 gene), (**H***) AtSOS3* (the salt overly sensitive 3 gene) and (**I**,**J**)*AtMAPK3/6* (the mitogen-activated protein kinase 3/6 genes). *AtActin2* served as an internal control for expression normalization. Forward and reverse primers for all tested genes are listed in Table S1. Bars (± SD) represent the means of three to five individual plants; a‒f indicate significant differences between treatments (*p* < 0.05).

To further investigate the interactions of salt-induced ethylene, eATP, H_2_O_2_, and [Ca^2+^]_cyt_, we used qPCR to evaluate the expression of some salt-responsive genes in Col-0 and *etr1-1* roots after treatment with ethylene, eATP, and their inhibitors with or without NaCl stress; namely, the expression of *AtSOS1* (encoding the PM Na^+^/H^+^ antiporter, Figure 7A), *AtAHA1* (responsive for the PM H^+^-pumps, Figure 7B), *AtRbohF* (function for the H_2_O_2_ signaling pathway), *AtEIN3*, *AtEIL1*, *AtETR1* (involved in the ethylene signaling pathway, Figure 7C–E), *AtSOS2/3* (responsive for the Ca^2+^ signaling pathway, Figure 7G,H), and *AtMAPK3/6* (regulation of the eATP signaling pathway, Figure 7I,J). qPCR revealed that in the absence of short-term salt treatment, the application of exogenous ACC to Col-0 plants remarkably upregulated the expression of *AtSOS1/3*, *AtRbohF*, *AtEIN3*, *AtEIL1*, and *AtETR1*, whereas the expression of *AtMAPK3/6* was not significantly different (Figure 7A,C–F,H). These findings suggest that the ethylene signaling cascade could upregulate the PM Na^+^/H^+^ antiporter and its downstream signaling pathways, such as those involving H_2_O_2_ and Ca^2+^, but there were no clear influences on the eATP signaling cascade in Col-0 roots. In addition, NaCl boosted the expression of a sequence of NaCl-related genes, such as *AtSOS1/3*, *AtEIN3*, *AtEIL1*, *AtETR1*, and *AtMAPK6* (Figure 7A,C–E,H,J). These results indicate that the PM Na^+^/H^+^ antiporter, [Ca^2+^]_cyt_, ethylene signaling, and eATP signaling responded remarkably to NaCl stress. NaCl and ACC upregulated the expression of all tested genes with the exception of *AtMAPK3* (Figure 7A–I), which further corroborated that ethylene signaling does not mediate the eATP signaling pathway. Moreover, eATP, a novel player in the salt response of *Arabidopsis*, enhanced the transcription of *AtSOS1/2/3*, *AtAHA1*, *AtMAPK3/6*, *AtEIN3*, *AtEIL1*, *AtETR1*, and *AtRbohF*, which implies that the eATP signaling cascade exhibits the most rapid response to salt injury (Figure 7). In terms of *etr1-1*, eATP increased *AtEIL1* and *AtETR1* expression in the absence of NaCl stress. These results suggest that eATP acts as a regulator of ethylene signaling to some extent (Figure S2).

## 3. Discussion

### 3.1. Ethylene and eATP Contribute to the Activation of the PM Na^+^/H^+^ Antiport System in Salt-Stressed Plants

#### 3.1.1. Na^+^ Homeostasis

Salinity poses a fatal threat to higher plants by effects on growth and metabolism [42], e.g., by shortening root length and ion leakage (Figure 1AB and Figure S1). To counteract this stress, the maintenance of K^+^/Na^+^ homeostasis is critical in determining the salt-endurance capacity of salinized plant species [1,7,43,44]. Under short-term saline conditions, the annual model plant species, *Arabidopsis*, e.g., Col-0, *etr1-1*, and *ein3-1eil1-1*, exhibited remarkable Na^+^ restriction and subsequent H^+^ absorption compared to the salt-untreated group (Figure 2A–F). The opposing scenario was observed when salt-elicited Na^+^ efflux and H^+^ influx were simultaneously restrained upon involvement of amiloride (a specific inhibitor of the PM Na^+^/H^+^ antiporter system) or sodium orthovanadate (a known inhibitor of the PM H^+^-ATPase) (Figure 2A‒D). These results indicate that Na^+^ extrusion and H^+^ uptake in salinized Col-0, *etr1-1*, and *ein3-1eil1-1* roots results from the activation of PM Na^+^/H^+^ antiporters and H^+^-ATPase. These results are in accordance with previous studies of multifarious woody and herbaceous plant species, i.e., *Populus euphratica* [6], *Bruguiera gymnorhiza* [7], *Aegiceras corniculatum* [8], and *G. uralensis* [10]. In addition, Na^+^ extrusion in salt-treated *Arabidopsis* was probably due to the activation of Ca^2+^‒SOS signaling pathways, in which elevation of cytosolic Ca^2+^ would promote the activity of the PM Na^+^/H^+^ antiport system, i.e., the Na^+^/H^+^ antiporters and H^+^-ATPase, to resist Na^+^ toxicity in the cytosol (Figure 2J‒L) [9,45,46]. It is worth noting that upon exposure to salt stress, Col-0 exhibited greater Na^+^ efflux, H^+^ influx, and Ca^2+^ influx than ethylene-insensitive mutants (Figure 2A–C,G–L). These findings suggest that the loss of ethylene signaling pathway function could weaken the salt tolerance in plants under high-NaCl conditions. In salinized *Arabidopsis*, ethylene and eATP enhanced Na^+^ efflux, whereas specific pharmacological agents for the involved molecules, i.e., PZA and suramin, could suppress the salt-elicited Na^+^ efflux (Figure 4A,B and Figure 5A,B). The above results suggest that both ethylene and eATP play crucial roles in upregulating the PM Na^+^/H^+^ antiport system under saline conditions. In the past, ethylene has been demonstrated to be involved in the maintenance of Na^+^ homeostasis, probably via promotion of the activity of PM H^+^ pumps in salt-stressed *Arabidopsis* roots [47]. Moreover, P2-like receptor antagonists, i.e., suramin or PPADS, could reduce Na^+^ exclusion, as *AHA1* expression was blocked in poplar cells [30,44].

qPCR assays showed that NaCl resulted in increased levels of *AtSOS1* transcripts and activation of the PM Na^+^/H^+^ antiport system in *Arabidopsis* roots (Figure 7A). Furthermore, we found that the expression of *AtSOS1* and *AtAHA1* was enhanced by exogenous ATP-Na_2_ or ACC treatment during short-term NaCl stress (Figure 7A,B), which is in line with our NMT data. These results presumably indicate that eATP or ethylene respond positively in terms of regulating the expression of genes for PM Na^+^/H^+^ antiporters and PM H^+^ pumps in salinized roots (Figure 7A,B). In our previous study, eATP, as a novel player in salt signaling, was demonstrated to significantly upregulate the expression of *GuSOS1* and *GuAHA1* in salt-treated licorice roots [10]. In addition, similar results were found for the poplar species *P. euphratica* [9,30]. Ethylene was required for the activation of PM H^+^-ATPase, resulting from upregulation of *AtAHA1* transcription in NaCl-stressed roots (Figure 7B), which is in accordance with the results of a previous study by Li et al. [47].

#### 3.1.2. K^+^ Homeostasis

The maintenance of K^+^ homeostasis in salinized shoots and roots is crucial for plant salt tolerance [48]. Under short-term NaCl treatment, Col-0, *etr1-1*, and *ein3-1eil1-1* collectively exerted obvious K^+^ leakage; furthermore, these phenomena were inhibited by TEA but promoted by sodium vanadate (Figure 3E,F). These effects are consistent with previous studies that show massive K^+^ loss under the regulation of depolarization-activated channels, e.g., KORCs and NSCCs, in the presence of NaCl stress [7,44,49]. We also found that exogenous ethylene and eATP were dramatically beneficial in promoting intracellular K^+^ retention (Figure 4C and Figure 5C). However, the application of PZA or suramin caused significant promotion of K^+^ leakage (Figure 4D and Figure 5D). Presumably, these results are attributed to the activation of PM H^+^ pumps, which play an important role in blocking K^+^-permeable channels. In our previous study, salt-elicited K^+^ release decreased in the presence of eATP in *K. obovata*, *A. corniculatum* [8], and *G. uralensis* [10]. Our qPCR assays showed that ethylene was required for the activation of PM H^+^ pumps through upregulation of *AtAHA1* transcription in NaCl-stressed roots. However, it is interesting that eATP application did not increase the salt-responsive induction of *AtAHA1* (Figure 7B). This probably implies that eATP might have an effect on a homolog of *AtAHA1*, which plays a similar role to *AtAHA1* in mediating proton efflux in the presence of salinity. Indeed, eATP promoted root skewing of *Arabidopsis*, which required energy from the predominant plasma membrane H^+^-ATPase, AHA2 [50].

### 3.2. Ethylene Is Involved in eATP, H_2_O_2_, and [Ca^2+^]_cyt_ Modulation of K^+^/Na^+^ Homeostasis in Response to Salinity Stress

Extracellular ATP, an emerging “pioneer signal”, is considered to contribute to salt-stress acclimation by provoking stress signaling pathways. Choi et al. (2014) reported that eATP binds PM P2K1 receptors, resulting in the execution of downstream functions, i.e., triggering of ROS and [Ca^2+^]_cyt_ [30,34,36]. In this study, eATP significantly shortened Col-0 hypocotyls under the no-salt conditions (Figure 1C,D). However, this effect of eATP on hypocotyl elongation was abolished in *etr1-1* and *ein3-1eil1-1* loss-of-function mutants, implying that eATP acts via ethylene response pathways. In previous studies, it has been suggested that eATP could promote activity of the ethylene signaling pathway, such as through upregulating the transcripts of biosynthetic and downstream responsive genes [51,52]. Accordingly, exogenous ATP increased the expression levels of *EIN3* and *EIL1* in Col-0, whereas the induction of *EIN3* by eATP was virtually indiscernible to that of the *etr1-1* mutant (Figure S2). In the presence of NaCl, eATP alleviated NaCl-induced growth retardation and reduced ion leakage but had no effects on *etr1-1* and *ein3-1eil1-1* mutants (Figure 1A,B and Figure S1). Under salt stress, eATP elevated Na^+^ extrusion and suppression of K^+^ leakage was impaired in *etr1-1* and *ein3-1eil1-1* (Figure 5A,C). Moreover, the NaCl-induced expression of *AtEIN3* and *AtEIL1* was restrained by the P2 receptor antagonist suramin (Figure 7C‒E). These findings indicate that the salt-induced ethylene component was probably elicited via the eATP signaling pathway and, thus, contributed to downstream events collectively in response to saline conditions. It is notable that MAPK3 and MAPK6, which are of particular importance in the eATP signaling pathway [34], alleviate salt sensitivity through maintenance of ethylene homeostasis in tobacco [53] and rice [54].Thus, it can be inferred that in salt conditions, extracellular ATP promotes the ethylene signaling pathway through MAPK modules.

It is well known that ethylene is an indispensable component in the response to salt stress of various plant species. We found that crosstalk between salt-induced messenger molecules, i.e., ethylene, eATP, H_2_O_2_, and cytosolic Ca^2+^, occurs in the mediation of ionic homeostasis under saline conditions. To be specific, ethylene was not only triggered by eATP (Figure 1C,D, Figure 5, and Figure 7C–E) but could also give rise to intracellular H_2_O_2_- and Ca^2+^-specific fluorescent accumulation in salt-exposed plants (Figure 2). In our previous study, eATP was found to interact with H_2_O_2_ and cytosolic Ca^2+^ to modulate ionic homeostasis in *P*. *euphratica* [30] and mangrove species [8] under salt stress. Moreover, in salinized *G. uralensis*, eATP stimulated the transcription of *GuRbohD*, *GuSOS3*, and *GuCIPK*, which were necessary to generate cytosolic ROS and Ca^2+^‒SOS signaling pathways, and then functioned together with these signaling molecules in the activation of PM Na^+^/H^+^ antiporters and H^+^-ATPase [10]. In the present study, exogenous ACC treatment dramatically upregulated the expression of *AtRbohF* and *AtSOS2*/3 under salt-stress conditions, which directly contributed to H_2_O_2_ and Ca^2+^ signaling pathways, respectively (Figure 7F–H). In line with our results, ethylene signaling cascades positively enhanced salt tolerance via upregulation of *AtRbohF* expression, which is responsible for vasculature tissue-specific ROS biosynthesis [55]. On the other hand, it is suggested that there is crosstalk between ethylene and salt-induced H_2_O_2_, wherein ethylene acts as a downstream event of ROS to synergistically regulate physiological and transcriptional changes in plant K^+^ homeostasis [41]. Moreover, Quan et al. demonstrated that NaCl-triggered SOS2 could not only phosphorylate EIN3 but might also boost EIN3 transcriptional activity or protein stability [38], which indicates regulation of the ethylene pathway by cytosolic Ca^2+^ signaling. On the contrary, ethylene was shown to evoke cytosolic Ca^2+^ by regulating PM Ca^2+^-permeable channels [56], so combined with the results of our qPCR assay of *AtSOS1/2*/3 (Figure 7A,G,H), it might be inferred that ethylene upregulated Ca^2+^ under salinity stress.

**Figure 8:**
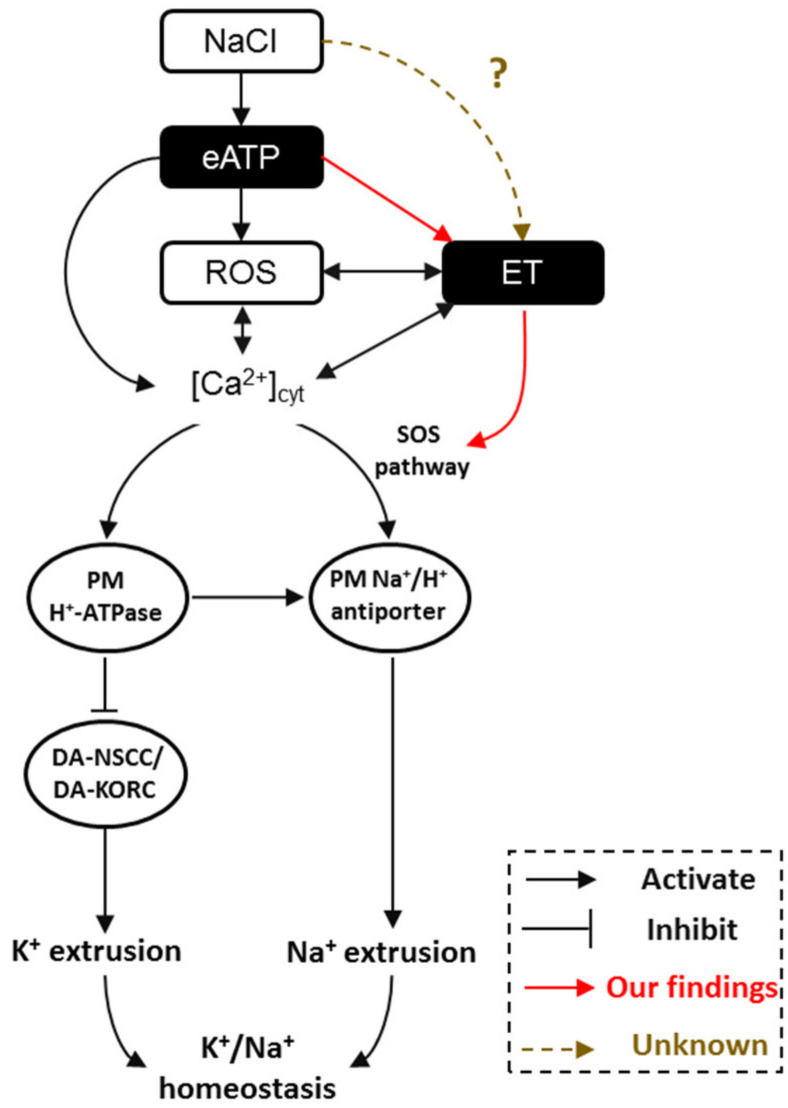
The proposed model of a multiple salt-signaling network involving eATP, ethylene, H_2_O_2_, and [Ca^2+^]_cyt_ in mediating K^+^/Na^+^ homeostasis in *Arabidopsis*. When sodium ions (Na^+^) are sensed by *Arabidopsis* roots, extracellular ATP is produced [10,30], resulting in ROS [8,30] and [Ca^2+^]_cyt_ [8,10] accumulation. Meanwhile, eATP activates ethylene signaling via upregulation of the expression of *AtEIN3*, *AtEIL1*, and *AtETR1* (a novel finding in the present study). After that, interactions between ROS and [Ca^2+^]_cyt_ [8,29,30,32,33,46,56], ROS and ethylene [25,41,55], and ethylene and [Ca^2+^]_cyt_ [38,39,40,56] regulate the PM Na^+^/H^+^ antiport system, including Na^+^/H^+^ antiporter (via SOS pathway) and H^+^-ATPase (H^+^ pumps). Furthermore, ethylene directly activates the SOS signaling pathway by increasing the transcription of *AtSOS1/2/3* (a novel finding in the present study). Consequently, the PM Na^+^/H^+^ antiporter helps to exclude the excess Na^+^ and H^+^-ATPase inhibits DA-KORCs/DA-NSCCs to limit cytosolic K^+^ leakage, namely by maintaining K^+^/Na^+^ homeostasis in salinity stress. ET: ethylene; PM: plasma membrane; DA-KORCs: depolarization-activated K^+^ outward rectifying channels; DA-NSCCs: depolarization-activated non-selective cation channels; ROS: reactive oxygen species.

In considering all the evidence together, we now know that interactions exist between eATP, ethylene, H_2_O_2_ (ROS), and Ca^2+^‒SOS signaling pathways in modulating plant salt tolerance. Briefly, under salt stress, cells suffer injury from invading Na^+^ and K^+^ efflux. ATP is then released to the extracellular matrix and binds purinergic P2K1 receptors. Activated eATP signaling stimulates the production of cytosolic H_2_O_2_ and Ca^2+^, and elicits the ethylene signal pathway. These signaling components engage in crosstalk to promote the H^+^-pump and SOS transport system to maintain intracellular K^+^/Na^+^ homeostasis (Figure 8).

## 4. Materials and Methods

### 4.1. Plant Materials and Culture Conditions

*Arabidopsis thaliana* Col-0, *etr1-1*, and an *ein3* and *eil1* double mutant (*ein3-1eil1-1*) were used in the study. Homozygous lines of *etr1-1* and *ein3-1eil1-1* were obtained from Hongwei Guo and all mutant seeds were in a Col-0 background [57]. Surface-sterilized seedlings were transferred onto round agar plates containing 1/2 MS (Sigma-Aldrich, St. Louis, MO, USA) basal salt medium, 1% sucrose, and 0.8% agar (pH 5.7‒5.8). To enhance uniform plant germination, the culture plates were incubated at 4 °C for 2‒3 days in the dark and then placed vertically at 23 °C under a light intensity of 200 μmol m^−2^ s^−1^. The photoperiod cycle was 16 h of light and 8 h of dark.

### 4.2. Phenotypic Identification and Physiological Experiments

The phenotypic identification in this study can be divided into three parts. Part 1: To confirm the effect of eATP on hypocotyl change belonging to ethylene triple response, Col-0 seeds were sown on 1/2 MS medium with dark treatment; after germination, the seedlings were screened by a stereoscope (Stemi 2000-C, Zeiss, Oberkochen, Germany) and the hypocotyl length was calculated. Part 2: Col-0, *etr1-1*, and *ein3-1eil1-1* were sown on a 1/2 MS agar plates without or with 100 mM NaCl for phenotypic analysis. Briefly, no fewer than 50 seeds of Col-0 and ethylene-insensitive mutants were sown onto each plate containing 1/2 MS medium subjected to 50 or 100 mM NaCl or not. Part 3: To determine the effect of eATP on the phenotype of ethylene-insensitive mutants, we added ATP-Na_2_ (a donor of extracellular ATP, 300 μM, Sigma-Aldrich, St. Louis, MO, USA) on the salt plates (50 or 100 mM) based on a 1/2 MS medium. Each physiological experiment on a phenotype needed to be replicated at least three times. Root length and relative electrolyte leakage were computed after a week since the transfer to the light. The relative electrolyte leakage of the seedlings was evaluated with the method reported in Peever and Higgins [58].

### 4.3. Salt, Antagonist, and Antagonist Treatments for NMT and qPCR Assays

Based on the culture conditions above, hydroponic-equilibrated seedlings, e.g., Col-0, *etr1-1*, and *ein3-1eil1-1*, were exposed to 0 or 100 mM NaCl solution for 12 h. To determine the interactions of ethylene, eATP, Ca^2+^, and H_2_O_2_ in regulating ionic homeostasis, two series of experiments were designed and carried out, as depicted below.

#### 4.3.1. Series 1: Agonist Treatments

Exogenous chemicals ATP-Na_2_ (300 μM) and 1-aminocyclopropanecarboxylic acid (ACC, a donor of ethylene, 10 μM, Sigma-Aldrich, St. Louis, MO, USA) were added to illuminate the relationship between eATP and ethylene in terms of regulating K^+^/Na^+^ homeostasis in young plants. This involved different amounts of agonists, in the presence or absence of NaCl (100 mM), as applied in a 1/2 MS nutrient solution, which was used for control and treatment plants.

We also measured the changes in the transcript levels of NaCl-resistant genes in *Arabidopsis* roots after the application of agonists with or without 12 h of salt treatment, e.g., ATP and ethylene. Specifically, the genes involved in K^+^/Na^+^ homeostasis and salt-induced signaling were *AtAHA1*, the PM H^+^-ATPase; *AtSOS1*, the PM Na^+^/H^+^ antiporter gene; *AtETR1*, the ethylene response 1 gene; *AtEIN3,* the ethylene-insensitive 3 gene; *AtEIL1*, the EIN3-like 1 gene; *AtSOS2/3*, the salt overly sensitive 2/3 genes; *AtRbohF*, the respiratory burst oxidase homolog protein F gene; and *AtMAPK3/6*, the mitogen-activated protein kinase 3 and 6 genes.

#### 4.3.2. Series 2: Antagonist Treatments

The pharmacological effects of several PM channel inhibitors were tested in salt-treated *Arabidopsis* seedlings, i.e., amiloride (a PM Na^+^/H^+^ antiporter blocker, 50 μM, Sigma-Aldrich, St. Louis, MO, USA) and sodium orthovanadate (a specific inhibitor of the PM H^+^-ATPase, 500 μM, Sigma-Aldrich, St. Louis, MO, USA) were used to inhibit the PM Na^+^/H^+^ antiport system, while tetraethylammonium chloride (TEA, a typical K^+^ channel inhibitor, 50 μM, Sigma-Aldrich, St. Louis, MO, USA) was used to block the salt-induced K^+^ efflux [6,7,10].

The effects of specific antagonists (100 μM PZA, Sigma-Aldrich, St. Louis, MO, USA and 300 μM suramin, Sigma-Aldrich, St. Louis, MO, USA, two inhibitors of the PM ethylene receptor and PM P2-like, respectively) on control and short-term salt-treated *Arabidopsis* were also evaluated in this study [30,59]. After the treatment of salt and antagonists, steady-state ion fluxes of the meristematic zone were promptly recorded. Next, the salt-elicited abundances of *AtAHA1*, *AtSOS1*, and some salt-related genes were examined in young roots with or without antagonist treatments.

### 4.4. Recording Net Ion Fluxes

A professional ion flux measurement system, a non-invasive micro-test technique (NMT; NMT-YG-100, Younger, Amherst, MA, USA), was used to determine the net K^+^, Na^+^, H^+^, and Ca^2+^ fluxes in *Arabidopsis* roots after 12 h of agonist and NaCl treatments. The preparation and calibration of ion-selective electrodes were described in previous studies [6,7,8,45]. The electrode calculations with Nernstian slopes >52 mV/decade were then used for K^+^, Na^+^, and H^+^ fluxes and Nernstian slopes >26 mV/decade for Ca^2+^ flux. All the net ionic fluxes were evaluated by Fick’s law of diffusion (see Equation (1)):

(1) $$J\;=\;-D\;(\frac{\mathrm{dc}}{\mathrm{dx}}),$$

where *J* stands for the ion flux in the x direction, *D* is the ion diffusion constant, and dc/dx represents the ion concentration gradient in the tested medium.

The 12-h NaCl-stressed seedlings were simultaneously exposed to agonists (ATP-Na_2_ and ACC) or antagonists (suramin and PZA). After that, the treated young root segments with apices of 2‒3 cm were collected and rinsed with redistilled water two to three times. Then, the roots were immediately equilibrated in fresh measuring solution (background: 0.1 mM NaCl, 0.5 mM KCl, 0.1 mM MgCl_2_, and 0.1 mM CaCl_2_ with a pH of 5.7 adjusted via choline and HCl) for 30 min to decrease the effect of salt release on flux recording.

After equilibration, the premeasured roots were transferred to the bottom of a glass lab dish containing 10‒15 mL of a specific measuring solution. To acquire the most accurate data, we chose test points in the meristem zone (500 μm from the root apex) as our net ion flux measurement field [7,8,10]. Each root needed to be continuously recorded for 15‒20 min along the root axis.

### 4.5. Quantitative Real-Time PCR Assays of Gene Expression

We used qPCR assays (Bio-Rad CFX96 Touch^TM^ real-time PCR detection system, Berkeley, CA, USA) to detect changes in the expression of some salt-related genes. TRIzol solution (Invitrogen, Carlsbad, CA, USA) was used to isolate the total RNA from Col-0 roots with or without short-term (12 h) salt treatment. After removal of DNA by DNase I (Promega, Madison, WI, USA) treatment for 30 min, aliquots of purified RNA (1 μg) as templates, M-MLV reverse transcriptase (Promega, Madison, WI, USA), and oligo(dT) primers (Takara, Dalian, China) were used for first-strand cDNA synthesis. The *Arabidopsis* β-actin 2 gene (*AtActin2*) was used as the internal control for normalization. Details of specific forward and reverse primers for *AtSOS1/2/3*, *AtAHA1*, *AtMAPK3/6*, *AtETR1*, *AtEIN3*, *AtEIL1*, and *AtRbohF* used in the qPCR are listed in Table S1.

The amplification method was conducted according to Deng et al. and the calculation of gene expression was performed via the relative 2^−^^△△CT^ approach [60,61].

### 4.6. Density of Cytosolic H_2_O_2_ and Ca^2+^ in Root Cells

Endogenous H_2_O_2_ and [Ca^2+^]_cyt_ in *Arabidopsis* roots were specifically detected using a green fluorescent probe, H_2_DCF-DA (50 μM, Eugene, Shanghai, China) and an orange fluorescent probe, Rhod-2 AM (2 μM, Biotium, San Francisco, USA), respectively [6,10,30]. Briefly, 10 μM ACC was added to 1/2 MS liquid medium supplemented with 0 or 100 mM NaCl for 30 min. Next, the roots were transferred to a fresh MES-KCl loading buffer (5 mM, pH 5.7) containing H_2_DCF-DA or Rhod-2 AM, respectively, in the dark to observe the intracellular H_2_O_2_ or [Ca^2+^]_cyt_ fluorescence after 1 h of incubation, when vigorous fluorescence could typically be seen [10]. After washing with 1/2 MS liquid medium 3‒4 times, the levels of endogenous H_2_O_2_ and [Ca^2+^]_cyt_ in roots were visualized by confocal microscopy prior to fluorescence intensity calculation. The confocal parameters for determination of cellular signal contents were as follows: excitation at 488 nm for H_2_DCF-DA and 543 nm for Rhod-2 AM, emission at 510‒530 nm for H_2_DCF-DA and 570‒590 nm for Rhod-2 AM, and frame 512 × 512 [6,10,30].

### 4.7. Data Analysis

Net ion fluxes were calculated using the data processing software JCal v. 3.0. Fluorescence intensity was counted using the image processing software Image-Pro Plus v. 6.0. All mean data were subjected to ANOVA for statistical treatments and denoted as the means ± SE in SPSS v. 19.0. Differences were considered statistically significant when *p* < 0.05, unless otherwise stated.

## 5. Conclusions

Our findings suggested that in salt-treated *Arabidopsis*, ethylene significantly assists in maintaining K^+^/Na^+^ homeostasis via activation of the PM Na^+^/H^+^ antiport system (Na^+^/H^+^ antiporters and H^+^-ATPase), including by enhancing Na^+^ extrusion, restricting K^+^ leakage, and upregulating the expression of *AtSOS1* and *AtAHA1*. Moreover, NaCl-induced ethylene is triggered by eATP, which then mediates downstream signaling cascades, e.g., H_2_O_2_ and cytosolic Ca^2+^, and finally interacts with these signaling molecules to ultimately activate the PM Na^+^/H^+^ antiport system (Na^+^/H^+^ antiporters and H^+^-ATPase) in response to salt adversity.

## Supplementary Materials

Supplementary materials can be found at https://www.mdpi.com/1422-0067/21/22/8683/s1. Table S1: Primers of a subset of salt-responsive genes for quantitative real-time PCR. Figure S1: Down-regulation of extracellular ATP results in alterations of relative electrolyte leakage upon Col-0, *etr1-1*, and *ein3-1eil1-1* plants in the presence or absence of salinity. Seeds were sown on 1/2 MS medium, after germination, plants were transferred to media subjected to 50 or 100 mM NaCl in the presence or absence of ATP-Na_2_ (300 μM), and plants were cultured in 1/2 MS NaCl-free (0 mM NaCl) medium as control. The relative electrolyte leakage was tested after a 10-day development. Each column (± SD) represents the mean of five to six individual plants and letters such as a, b, and c denote a significant difference between different treatments, *p* < 0.05). Figure S2: Effects of ACC and eATP on some salt-responsive genes transcription of *etr1-1* roots under salt stress. Roots were treated by ATP-Na_2_ (300 µM) or 100 mM NaCl for 12 h. Quantitative RT-PCR results show the relative transcript abundance of homolog genes in *etr1-1*, such as *AtSOS1* (the PM salt overly sensitive 1/ Na^+^/H^+^ antiporter), *AtAHA1* (the PM H^+^-ATPase), *AtSOS2* (the salt overly sensitive 2 gene), *AtSOS3* (the salt overly sensitive 3 gene), *AtRbohF* (the respiratory burst oxidase homolog protein F gene), *AtEIN3* (the ethylene insensitive 3 gene), *AtEIL1* (the EIN3-like 1 gene), *AtETR1* (the ethylene response 1 gene), and *AtMAPK3/6* (the mitogen-activated protein kinase 3/6 genes). *AtActin2* served as an internal control for expression normalization. Forward and reverse primers for all tested genes are listed in Table S1. Bars (± SD) represent the means of three to five individual plants; gray lines represent internal controls (value, 1); letters (a and b) indicate significant differences between treatments (*p* < 0.05).
