# Supplementary material for: A Salt-Signaling Network Involving Ethylene, Extracellular ATP, Hydrogen Peroxide, and Calcium Mediates K+/Na+ Homeostasis in Arabidopsis
Source: Int J Mol Sci. 2020 Nov 17;21(22):8683. doi: 10.3390/ijms21228683 (PMC7698765; doi:10.3390/ijms21228683)
Supplement: Supplementary file 1 [file ijms-21-08683-s001.pdf]

International Journal of
Molecular Sciences

MDPI

Table S1. Primers of a subset of salt-responsive genes for quantitative real-time PCR.

Gene	Primer Sets	Sequence（5′ to 3′）
AtACT2	Forward	GGTAACATTGTGCTCAGTGGTGG
	Reverse	AACGACCTTAATCTTCATGCTGC
AtAHA1	Forward	GACAGGATTGTGATATTTGGCC
	Reverse	CGGAGGTCGATTATCACCATTA
AtSOS1	Forward	ATTTTGATGCAGTCAGTGGATG
	Reverse	GCAAGCAGATTCTAGTCTTTCG
AtSOS2	Forward	GCGAACTCAATGGGTTTTAAGT
	Reverse	CTTACGTCTACCATGAAAAGCG
AtSOS3	Forward	CCGGTCCATGAAAAAGTCAAAT
	Reverse	CTCTTTCAATTCTTCTCGCTCG
AtRbohF	Forward	TATTGGAGACCATCTTGCTTGT
	Reverse	CGTTAAAACCGGTTAGTCGATC
AtETR1	Forward	TTCCCCGACATTCAAATTTCAC
	Reverse	GTGGATTTGTCAGTGTTACCAC
AtEIN3	Forward	CTGCAGATCACAACAACTTTGA
	Reverse	CATCCATCGTTCCTACTACTCC
AtEIL1	Forward	TTGAAGAAAGCTTGGAAAGTCG
	Reverse	TTTGATTGCCTCACAAGCTTAC
AtMAPK3	Forward	TGACAGAATGTTGACGTTTGAC
	Reverse	GATTGAGTGCTATGGCTTCTTG
AtMAPK6	Forward	AGAATATTCCGGCGACTCTTAG
	Reverse	ATTCATAGCCGAACAAACGATG

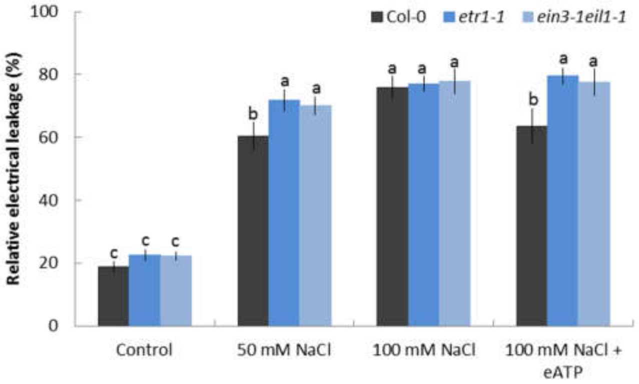

Figure S1. Down-regulation of extracellular ATP results in alterations of relative electrolyte leakage upon Col-0, *etr1-1*, and *ein3-1eil1-1* plants in the presence or absence of salinity. Seeds were sown on 1/2 MS medium, after germination, plants were transferred to media subjected to 50 or 100 mM NaCl in the presence or absence of ATP-Na_2 (300 μM), and plants were cultured in 1/2 MS NaCl-free (0 mM NaCl) medium as control. The relative electrolyte leakage was tested after a 10 day development. Each column (± SD) represents the mean of five to six individual plants and letters such as a, b, and c denote a significant difference between different treatments, $p < 0.05$).

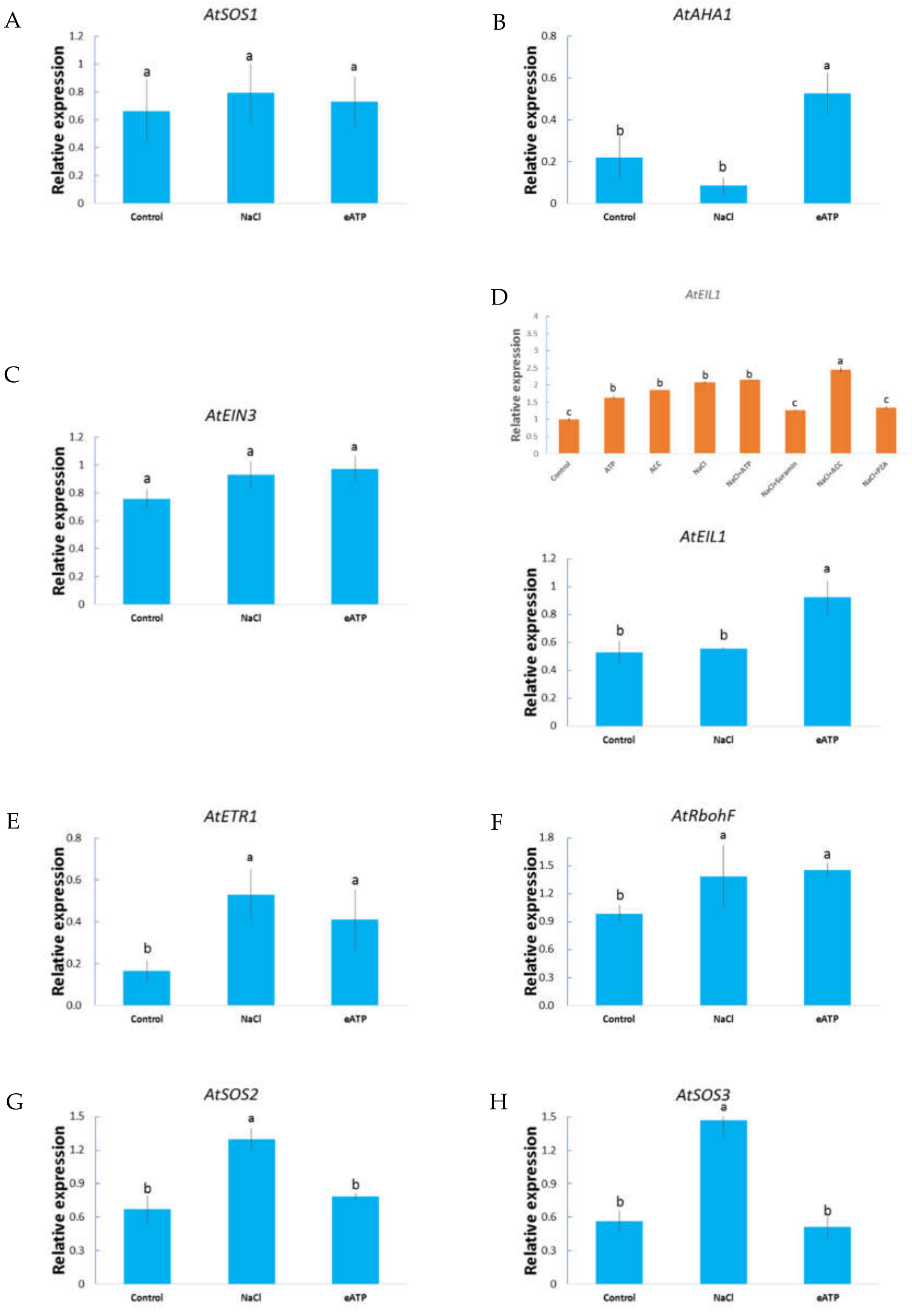
A
AtSOS1
Relative expression
Control
NaCl
eATP
B
AtAHA1
C
AtEIN3
D
AtEIL1
E
AtETR1
F
AtRbohF
G
AtSOS2
H
AtSOS3

I

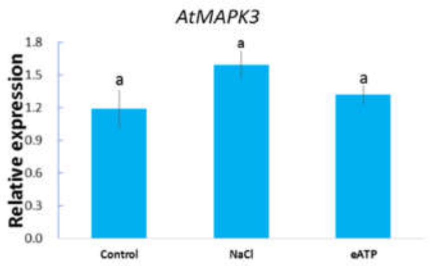

J

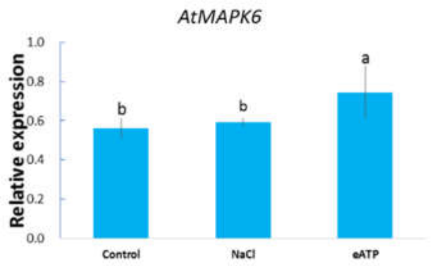

Figure S2. Effects of ACC and eATP on some salt-responsive genes transcription of *etr1-1* roots under salt stress. Roots were treated by ATP-Na_2 (300 μM) or 100 mM NaCl for 12 h. Quantitative RT-PCR results show the relative transcript abundance of homolog genes in *etr1-1*, such as (**A**) *AtSOS1* (the PM salt overly sensitive 1/ Na^+/H^+ antiporter), (**B**) *AtAHA1* (the PM H^+-ATPase), (**C**) *AtEIN3* (the ethylene insensitive 3 gene), (**D**) *AtEIL1* (the EIN3-like 1 gene), (**E**) *AtETR1* (the ethylene response 1 gene), (**F**) *AtRbohF* (the respiratory burst oxidase homolog protein F gene), (**G**) *AtSOS2* (the salt overly sensitive 2 gene), (**H**) *AtSOS3* (the salt overly sensitive 3 gene) and (**I**,**J**) *AtMAPK3/6* (the mitogen-activated protein kinase 3/6 genes). *AtActin2* served as an internal control for expression normalization. Forward and reverse primers for all tested genes are listed in Table S1. Bars (± SD) represent the means of three to five individual plants; gray lines represent internal controls (value, 1); letters (a and b) indicate significant differences between treatments ($p < 0.05$).
